# Reproducibility and Absolute Quantification of Muscle Glycogen in Patients with Glycogen Storage Disease by ^13^C NMR Spectroscopy at 7 Tesla

**DOI:** 10.1371/journal.pone.0108706

**Published:** 2014-10-08

**Authors:** Katja Heinicke, Ivan E. Dimitrov, Nadine Romain, Sergey Cheshkov, Jimin Ren, Craig R. Malloy, Ronald G. Haller

**Affiliations:** 1 Neuromuscular Center, Institute for Exercise and Environmental Medicine, Texas Health Presbyterian Hospital, Dallas, Texas, United States of America; 2 Department of Neurology and Neurotherapeutics, University of Texas Southwestern Medical Center, Dallas, Texas, United States of America; 3 Advanced Imaging Research Center, University of Texas Southwestern Medical Center, Dallas, Texas, United States of America; 4 Philips Medical Systems, Cleveland, Ohio, United States of America; 5 Department of Neurology, North Texas VA Medical Center, Dallas, Texas, United States of America; Institut de Recerca de la Santa Creu i Sant Pau, Spain

## Abstract

Carbon-13 magnetic resonance spectroscopy (^13^C MRS) offers a noninvasive method to assess glycogen levels in skeletal muscle and to identify excess glycogen accumulation in patients with glycogen storage disease (GSD). Despite the clinical potential of the method, it is currently not widely used for diagnosis or for follow-up of treatment. While it is possible to perform acceptable ^13^C MRS at lower fields, the low natural abundance of ^13^C and the inherently low signal-to-noise ratio of ^13^C MRS makes it desirable to utilize the advantage of increased signal strength offered by ultra-high fields for more accurate measurements. Concomitant with this advantage, however, ultra-high fields present unique technical challenges that need to be addressed when studying glycogen. In particular, the question of measurement reproducibility needs to be answered so as to give investigators insight into meaningful inter-subject glycogen differences. We measured muscle glycogen levels *in vivo* in the calf muscle in three patients with McArdle disease (MD), one patient with phosphofructokinase deficiency (PFKD) and four healthy controls by performing ^13^C MRS at 7T. Absolute quantification of the MRS signal was achieved by using a reference phantom with known concentration of metabolites. Muscle glycogen concentration was increased in GSD patients (31.5±2.9 g/kg w. w.) compared with controls (12.4±2.2 g/kg w. w.). In three GSD patients glycogen was also determined biochemically in muscle homogenates from needle biopsies and showed a similar 2.5-fold increase in muscle glycogen concentration in GSD patients compared with controls. Repeated inter-subject glycogen measurements yield a coefficient of variability of 5.18%, while repeated phantom measurements yield a lower 3.2% system variability. We conclude that noninvasive ultra-high field ^13^C MRS provides a valuable, highly reproducible tool for quantitative assessment of glycogen levels in health and disease.

## Introduction

Glycogen is a critical energy source in human skeletal muscle that is needed to support normal exercise capacity. Blocked glycogen breakdown in muscle glycogen storage diseases (GSD) causes severe exercise intolerance marked by premature fatigue with muscle contractures and rhabdomyolysis triggered by more vigorous exercise [1], [2]. In McArdle disease (MD, GSD type V) muscle glycogen breakdown is blocked due to muscle phosphorylase deficiency and in muscle phosphofructokinase deficiency (PFKD, GSD type VII) the utilization of muscle glycogen and blood glucose is prevented, which in both cases severely restricts oxidative and anaerobic metabolism [3], [4]. While the diagnosis in GSD V and VII may be achieved by genetic testing or enzymatic analysis of muscle tissue, potential limitations in these methods exist [5], [6]. In MD more than 100 mutations in the PGYM gene are known, and molecular screening may fail to identify less common pathogenic mutations or may identify genetic variations of uncertain significance. False positives in the diagnosis of PFKD are common due to the fact that phosphofructokinase is highly labile and enzymatic activity is easily lost by incorrect sample handling. Excess muscle glycogen is a hallmark of these disorders that is usually not recognized unless and until a muscle biopsy is performed. A sensitive means of assessing muscle glycogen noninvasively could facilitate diagnosis and could be used to monitor possible changes in glycogen concentrations with disease progression or in response to therapy. Carbon-13 magnetic resonance spectroscopy (^13^C MRS) has been reported as a noninvasive method for monitoring *in vivo* metabolites in skeletal muscle without the need for invasive tissue biopsies. Currently, however, the use of glycogen ^13^C MRS for diagnosis or monitoring therapy in metabolic myopathies has not achieved widespread research or clinical use, partly due to several technical limitations inherent to ^13^C MRS.

Notably, ^13^C MRS detects the concentration of glycogen directly by the natural abundance ^13^C C_1_ of glycogen. The low natural abundance of 1.1% of all carbons and low relative sensitivity of the ^13^C nucleus results in poor signal-to-noise ratio (SNR), [7], therefore, it is appealing to perform ^13^C MRS in ultra-high fields with the two major advantages of improved SNR and spectral resolution [8]. Concomitant with these advantages, ultra-high fields present unique technical challenges that need to be addressed when studying glycogen. In particular, it is unknown if the expected shorter T2 transverse relaxation time of glycogen at these high fields will result in fast signal decay and poor overall SNR. *In vivo* specific absorption rates (SAR) are also increased at ultra-high fields thus making the application of proton decoupling challenging. Furthermore, there are no studies to investigate the *in vivo* reproducibility of ^13^C MRS for glycogen detection, thus making quantitative results difficult to interpret. Finally, it is unknown if absolute quantification is feasible given that the radio frequency (RF) transmit field B_1_ coil profiles are more inhomogeneous at higher fields and the fact that the excitation and detection sensitivity may be effected by the variability of the subject-specific subcutaneous layer thickness.

In this study we evaluated the *in vivo* skeletal muscle glycogen content of healthy subjects and in patients with glycogen storage disease by performing noninvasive ^13^C MRS in a 7 Tesla magnet and investigated the feasibility of implementing this technology in the clinical evaluation of metabolic myopathies.

## Methods

### Subjects & Protocol

The study was approved by the Institutional Review Boards of the University of Texas Southwestern Medical Center and Texas Health Presbyterian Hospital, Dallas. Written informed consent was obtained from all subjects.

Muscle glycogen concentrations were determined by natural abundance ^13^C MRS in the calf muscle in three MD patients (1 female and 2 males, age 22±4 yrs, mean ± SD), one PFKD patient (male, 42 yrs) and 4 healthy control subjects (1 female and 3 males, age 42±3 yrs). There were no special dietary preparations but a minimum of 12 h rest prior to the measurements was required. In addition to these scans, in order to test reproducibility, one normal subject was scanned on three consecutive days, keeping the time of the day and the time period after a regular meal the same. Glycogen was also determined by diagnostic needle biopsy of the vastus lateralis muscle performed on eight MD patients (6 females and 2 males), one PFKD patient (male) and 4 healthy control subjects (1 female and 3 males). In two MD patients and one PFKD patient, muscle glycogen was measured by both MRS and biopsy.

Due to funding limitations, only local patients previously diagnosed by muscle needle biopsy, a standard method to establish the diagnosis of MD and related disorders, could be studied using MRS at 7T. These biopsies typically are performed on vastus lateralis muscle, whereas the 7T magnet in this study is optimized for the evaluation of calf muscles.

### Procedures & Measurements

#### ^13^C MRS

Spectra were acquired on a whole-body 7T scanner (Achieva, Philips Medical Systems, Cleveland, OH, USA) using a partial volume human calf coil, covering the main part of the gastrocnemius and soleus muscles, operating in quadrature for both proton (^1^H) and ^13^C. The ^1^H portion of the coil consisted of two overlapped rectangular loops, each loop having an 8.6 cm side length. These loops were curved and mounted on a half-cylinder former with a diameter of approximately 16 cm. The ^13^C portion of the coil consisted of a 9 cm side rectangular curved center loop and an overlapping 10.6 cm butterfly loop. Coupling between ^1^H and ^13^C coils was reduced by adding proton traps in the ^13^C coils. A segmented RF-shield surrounded the bottom and the sides of the coil. The ^13^C coil was closer than the ^1^H coil to the surface of the calf, and there was a 10 mm separation between the ^13^C and ^1^H coils. The final, usable external diameter of the coil was 14 cm. The coil had a small 10 ml vial of enriched 1,2-^13^C acetate fixed inside to serve as an external reference for absolute quantification. ^13^C spectra were acquired by averaging 1024 non-selective free-induction decays (FIDs) with a repetition time (TR) of 800 ms for a total scan time of 14 min. While the relatively long TR was mainly required in order to stay under the FDA-approved specific absorption rate (SAR) limits, it also helped with reducing a potential T1 longitudinal relaxation time bias in the evaluation of the glycogen/total creatine ratio. The excitation was centered at 110 ppm, some 10 ppm away from the glycogen signal, using a broad band (BW = 7812 Hz; 104 ppm) block pulse of duration 128 µs and RF-transmit field (B_1_) strength 186 µs. WALTZ-16 decoupling with a 12 µT B_1_ pulse centered at 4.1 ppm was used to simplify the spectra and enhance SNR. To avoid power limitations while preserving spectral resolution, the decoupling was performed only during the first 20% of the 256 ms acquisition time. In effect, the decoupling period aimed to cover the acquisition period during which the carbon signal was not completely decayed. No nuclear Overhauser effect (NOE) was applied. The potential NOE effects of the decoupling itself were negligible given that decoupling was performed only for about 50 ms out of a TR of 800 ms. Scans were acquired with BW 16 kHz and 4k points. Total scan time, including patient positioning, surveys and ^13^C MRS acquisition was about one hour. All subjects tolerated the scan well. Absolute quantification was achieved using the substitution method with a calibration phantom [9]. A 1-L cylindrical bottle was prepared with 100 mM oyster glycogen (molecular weight 180 g/mol), and 40 mM creatine (molecular weight 131.14 g/mol) dissolved in phosphate buffered saline (PBS) at a pH of 7.2. The phantom was scanned immediately after the subject, keeping all power calibration constant and with the same protocol as *in vivo*. No T1 correction was performed on the *in vivo* and phantom signals based on the assumption that glycogen T1 does not differ significantly *in vivo* and *in vitro* [10]. The issue of potential differences in the coil coverage between the *in vivo* and phantom cases was assessed by investigating if the coil sensitivity exceeded the volume of the single cylindrical 1-L volume phantom. First, the dimension along the main static magnetic field (B_0_) was checked; this was achieved by scanning two cylindrical phantoms placed with their long symmetry axis collinear. This dual phantom greatly exceeded the physical dimensions of the coil along the B_0_ field. It was noted that the signal was only 1% larger in the case of the dual-phantom as compared to a single-phantom. This potential difference in coil sensitivity coverage along the long axis was corrected for by multiplying the measured phantom signal intensities by 1.01. Second, calibration of the coil sensitivity along the short axis (anterior-posterior) and the closely related issue of the effects of varying subcutaneous fat thickness on the quantification of glycogen was addressed by performing a set of phantom measurements where the 1-L phantom was placed on fat-pads of different thicknesses and noting the change in the signal. The fat pads were made of small flexible bags filled with olive oil. Care was taken to make sure that the bags had uniform thickness along the phantom body without any noticeable folds or oil voids. Image scouts from the volunteers were used to measure the thickness of the subcutaneous layer and the data was corrected using the interpolated calibration sensitivity curve obtained from the phantom study. System stability and reproducibility of the measurements was established by a) preparing the calibration phantom two different times in order to judge reproducibility in having the same absolute concentrations, and b) by measuring glycogen, creatine and acetate signals three consecutive times, while keeping all the acquisition and scanner calibration parameters the same.

#### Muscle tissue biopsy and biochemical analysis

Muscle needle biopsies were taken from the mid-portion of the vastus lateralis. The 20 to 30 mg muscle samples were flash-frozen and stored in liquid nitrogen until analyzed. Glycogen content was measured in aliquots of 10% homogenates after boiling for 2 minutes followed by digestion with amyloglucosidase (Sigma A9228) for 2 hours at 37°C. After the addition of perchloric acid and centrifugation, glucose content was measured in aliquots of neutralized supernatants by an enzymatic spectrophotometric assay at 37°C as described by DiMauro et al. [11].

### Data analysis

MRS data processing was performed with DC correction, two-fold zero filling, exponential line broadening of 2 Hz, manual phasing and baseline correction. Integrals of the glycogen, total creatine and the reference acetate signals were drawn manually, keeping the intervals of integration consistent across all subjects. The glycogen integration was performed over a region of 10 ppm centered at 100.5 ppm in order to account for the broad baseline glycogen signal. Total creatine integration was performed in the region 157.5–159.5 ppm thus covering both creatine and phosphocreatine. For absolute quantification of glycogen and creatine, the phantom substitution method was used [9]. All quantities are reported in millimoles per liter (mM). To compare MRS data to biopsy data, muscle metabolite concentrations (mM) were converted to grams per kilogram wet weight (g/kg w. w.) using the constant of 1.1 kg·L^−1^ of muscle [12] and the molecular weights used for the phantom. Data are expressed as single values and/or mean ± standard deviation (SD).

## Results

**Figure 1:**
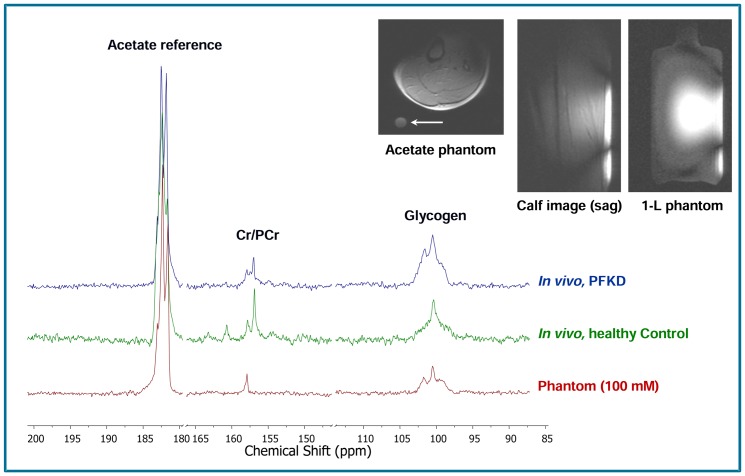
^13^C MRS spectra of glycogen and creatine (Cr)/phosphocreatine (PCr) in calf muscle showing glycogen accumulation in a patient with muscle phosphofructokinase deficiency (PFKD) compared with a healthy control subject and the reference quantification phantom at 7T. The observed doublet for the acetate reference is due to the C1-C2 coupling. An axial image of the calf showing the internal acetate phantom (white arrow). Sagittal images of the calf and the 1-L external calibration phantom show the similar approximate sensitivity of the coil.

**Figure 2:**
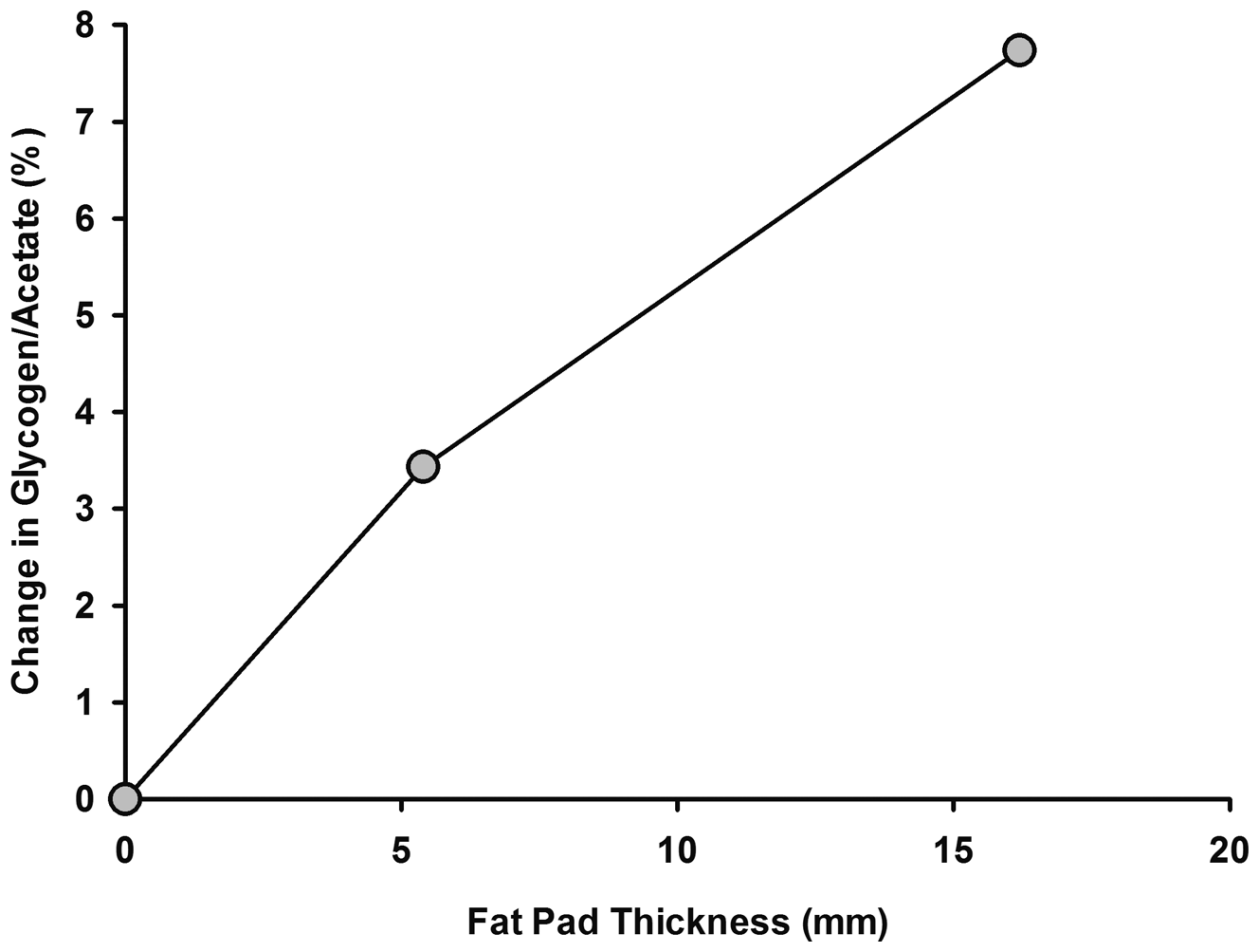
Correction factors for the glycogen/acetate ratio as a function of the thickness of the fat layer underneath the calibration phantom. All *in vivo* data were corrected for the measured thickness of the subcutaneous fat by using interpolated values from this figure. For most subjects this correction was in the order of 4−5%.

Typical ^13^C MRS spectra of muscle glycogen and total creatine in a PFKD patient, control subject and the reference phantom are shown in Fig. 1, along with typical calf muscle and reference phantom coverage images. The axial muscle T1-weighted image also shows (with white arrow) the circular cross section of the internal acetate calibration phantom, positioned inside the coil. As seen, similar sagittal excitation patterns were observed *in vivo* and in the 1-L external reference phantom, thus suggesting similar coil loading and sensitivities. Mean MRS signal intensities for glycogen and total creatine in mM are shown in Table 1. The values presented reflect a correction for the individual subcutaneous fat thickness, taken from interpolating the results from Fig. 2. For the most volunteers, the average subcutaneous fat thickness was in the order of 8–10 mm which would result in individual corrections of 4–5%.

**Table 1:** Skeletal muscle metabolites - ^13^C NMR spectroscopy.

Subjects	Glycogen (mM)	Total Creatine (mM)	Glycogen/Total Creatine
MD	192.5±15.1	85.6±22.5	2.3±0.6
PFKD	202.1	124.2	1.6
Controls	75.7±13.6	77.1±8.3	1.0±0.3

Values are expressed individually or group means ± SD. MD, McArdle disease; PFKD, phosphofructokinase deficiency; mM, millimoles per liter.

**Figure 3:**
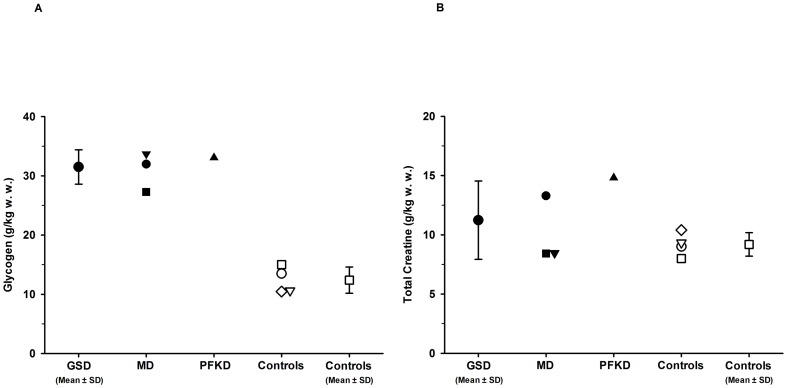
**Figure 3A–B**. Skeletal muscle glycogen (3A) and total creatine concentrations (3B) measured by ^13^C MRS. Values are expressed individually or group means ± SD. GSD, glycogen storage diseases; MD, McArdle disease; PFKD, phosphofructokinase deficiency; controls, healthy control subjects; g/kg w. w., grams per kilogram wet weight.

The mean muscle glycogen concentration (Fig. 3A) was 2.5-fold higher in GSD patients (31.5±2.9 g/kg w. w.) compared with control subjects (12.4±2.2 g/kg w. w.). There was no difference in muscle total creatine concentration (Fig. 3B) between the GSD patients (11.2±3.3 g/kg w. w.) and control subjects (9.2±1.0 g/kg w. w.). Muscle biopsy data showed a similar 2.5-fold higher muscle glycogen concentration in GSD patients (24.9±3.5 g/kg w. w.) compared with control subjects (10.0±1.7 g/kg w. w.). Similar values were achieved by both methods in the three GSD patients in whom muscle glycogen was assessed by both MRS (32.9±0.9 g/kg w. w.) and biochemistry (28.0±3.1 g/kg w. w.).

*In vivo* reproducibility and the corresponding intra-subject variability, as measured by the three identical studies on the same normal subject, showed a reported coefficient of variation 5.18% and 5.71% for glycogen and total creatine, respectively. This is about three times lower than the inter-subject coefficient of variation (17.83% for glycogen and 11.08% for total creatine, in normal subjects) thus making it possible to conduct meaningful comparisons of glycogen values among normal subjects. System stability and hardware measurement reproducibility, as assessed by measuring three times the signal from the concentration reference phantom, showed a standard deviation of 3.2%, and 9.0% for glycogen and creatine, respectively. In addition, the error in the absolute quantification of the metabolites introduced due to fluctuations in the preparation of the calibration phantom was assessed by preparing the phantom two separate times. This sample preparation error was measured at 5.5% and 2.45% for glycogen and creatine, respectively. Assuming independence of the errors, the total combined error of the final quantification is thus calculated to be 6.4% for glycogen and 9.3% for creatine. Comparison of the results of these repeated measurements *in vivo* and in phantoms show that, in terms of reproducibility, the hardware instabilities are smaller than the intra-subject variability, thus attesting to the meaningful comparison of *in vivo* data. However, in terms of absolute quantification, these results suggest that the limiting factor is the accuracy on the preparation of the calibration phantom. Greater care in preparing these calibration phantoms could thus decrease the error in the absolute quantification to the error in the hardware performance, which for our system is in the order of 3% for glycogen.

## Discussion

The present study is the first investigation utilizing ^13^C MRS at 7T for absolute quantification of muscle glycogen concentration in patients with GSD. A major focus of the study is to report on the reproducibility of these studies, both in vivo and in phantoms. The main findings are a 2.5-fold increase in muscle glycogen concentration in MD and PFKD patients in agreement with biochemical measured values, and the demonstration of the high reproducibility of *in vivo* ^13^C MRS at ultra-high fields as a noninvasive method for clinical diagnosis in GSD patients.

It has been shown that^ 13^C MRS is a valuable method for noninvasive assessments of glycogen levels in skeletal muscle in patients with MD and PFKD [13], [14], [15], and other GSD's [15], [16]. However, these studies were performed at lower magnetic fields with semiquantitative assessment of glycogen expressed as the ratio of glycogen to total creatine. In the present study, the semiquantitative values for glycogen/total creatine ratio in patients and controls were in line with the results found at high magnetic fields (4T), [15] but were below the values found at low magnetic fields (2T), [13], [14]. There has been controversy about the complete glycogen visibility by ^13^C MRS [17], and the discrepancy between the studies might be due to different magnetic field strength and the complex structure of glycogen, a branched polymer of glucose that varies in size and displays heterogeneity in localization within skeletal muscle cells and different muscle fiber types [18]. However, detection and reproducibility of glycogen content in skeletal muscle has been demonstrated earlier at high magnetic fields (4.7T), [19], and there was no difference found in glycogen concentration between calf and thigh muscle in healthy controls [15]. Furthermore, ^13^C MRS was validated in comparison with skeletal muscle biopsies by biochemical analysis in healthy subjects (R = 0.95), [20] and found to be at least as accurate as biopsy measurements (coefficients of variation MRS 4.3% vs. biopsy 9.3%). Using increased signal strength at 7T results in improved measurement accuracy [8] and reduced time required for glycogen assessment. The SNR for the C_1_ peak of glycogen using a phantom was increased by 60% at 7T compared with 3T for the same number of acquisitions. ^13^C MRS glycogen measurements in our study showed high *in vivo* reproducibility (5.18% for glycogen) with no overlap between the 2.5-fold higher glycogen concentrations in GSD patients compared with control subjects. Glycogen concentrations corresponded closely with the muscle needle biopsy values and absolute glycogen concentrations in control subjects were in agreement with results shown earlier at high magnetic field [20]. Overall, these findings strengthen confidence in the accuracy of our results.

The major advantages of *in vivo* ^13^C MRS include avoidance of invasive biopsy procedures hence providing the possibility of stress-less and painless assessment of glycogen in a larger muscle volume that would likely be less susceptible to local variations in muscle glycogen concentrations. The accurate quantification of muscle glycogen concentration at ultra-high fields allows assessment of physiological alterations and kinetic changes [9] and also can support the clinical diagnosis in GSD. Little is known regarding possible variations in glycogen storage in MD and PFKD, therefore, it would be beneficial to determine muscle glycogen concentrations in these disorders over time. Monitoring muscle glycogen levels in patients with blocked glycogen breakdown is important in defining the natural history of muscle glycogen levels as well as to determine possible effects of therapy. Both regular aerobic exercise and dietary modifications have been advanced as therapy in GSD V and VII [5], [6], [21], [22]. Fatiguing exercise in healthy humans promotes increased muscle glycogen deposition but the implications for glycogen levels in MD or PFKD are unknown [23]. Blood glucose is a critical alternative fuel in MD and adequate glucose availability as determined by hepatic glycogen stores and dietary carbohydrate, including the ingestion of simple sugars shortly before exercise have been shown to enhance exercise capacity in MD [24], [25]. However the implications of dietary carbohydrate for muscle glycogen concentrations are unknown.

In conclusion, muscle glycogen concentrations were 2.5-fold higher in patients with GSD type V and VII, and MRS measurements were consistent with the muscle needle biopsy results. This study clearly demonstrates the feasibility of accurately and reproducibly measuring glycogen in skeletal muscle using *in vivo* ^13^C MRS at ultra-high fields as a noninvasive method for facilitating clinical diagnosis and monitoring treatment effects in GSD patients.
